# Intrinsic Permeation and Anti-Inflammatory Evaluation of Curcumin, Bisdemethoxycurcumin and Bisdemethylcurcumin by a Validated HPLC-UV Method

**DOI:** 10.3390/ijms24076640

**Published:** 2023-04-02

**Authors:** Helen-Lissette Alvarado, David Limón, Ana-Cristina Calpena-Campmany, Mireia Mallandrich, Laura Rodríguez-Cid, Núria Aliaga-Alcalde, Arántzazu González-Campo, Lluïsa Pérez-García

**Affiliations:** 1 Departament de Farmàcia i Tecnologia Farmacèutica, i Fisicoquímica, Facultat de Farmàcia i Ciències de l’Alimentació, Universitat de Barcelona (UB), Av. Joan XXIII 27-31, 08028 Barcelona, Spain; 2 Institut de Nanociència i Nanotecnologia (IN2UB), Universitat de Barcelona (UB), 08028 Barcelona, Spain; 3 Departament de Farmacologia, Toxicologia i Química Terapèutica, Facultat de Farmàcia i Ciències de l’Alimentació, Universitat de Barcelona (UB), Av. Joan XXIII 27-31, 08028 Barcelona, Spain; 4 Institute of Materials Science of Barcelona (ICMAB-CSIC), Campus of the Universitat Autònoma de Barcelona, 08193 Bellaterra, Spain; 5 ICREA—Catalan Institution for Research and Advanced Studies, Passeig Lluis Companys 23, 08010 Barcelona, Spain

**Keywords:** curcuminoids, curcumin, bisdemethoxycurcumin, bisdemethylcurcumin, intrinsic ex vivo permeation, HPLC, method validation, histology, anti-inflammatory

## Abstract

Curcumin shows anti-inflammatory activity, and it has been widely investigated for neurodegenerative diseases, adjuvant treatment in AIDS and antitumor activity against different tumors, among other activities. The goal of this work was to evaluate the capacity of curcumin and its derivatives (bisdemethoxycurcumin and bisdemethylcurcumin) in preventing the irritant effects of topically applied xylol and to assess the intrinsic capacity of curcuminoids in permeating human skin by ex vivo permeation tests. Its secondary goal was to validate an HPLC method to simultaneously determine the curcuminoids in the samples from the ex vivo permeation studies and drug extraction from the skin. Curcuminoid quantification was performed using an RP-C18 column, at isocratic conditions of elution and a detection wavelength of 265 nm. The method was specific with a suitable peak resolution, as well as linear, precise, and accurate in the range of 0.195–3.125 μg/mL for the three curcuminoids. Bisdemethylcurcumin showed the greatest permeation through the human skin, and it was the curcuminoid that was most retained within the human skin. The anti-inflammatory activity of the curcuminoids was evaluated in vivo using a xylol-induced inflammation model in rats. Histological studies were performed to observe any changes in morphology at the microscopic level, and these three curcuminoids were found to be respectful within the skin structure. These results show that these three curcuminoids are suitable for anti-inflammatory formulations for dermal applications, and they can be properly quantified using HPLC-UV.

## 1. Introduction

**Figure 1:**
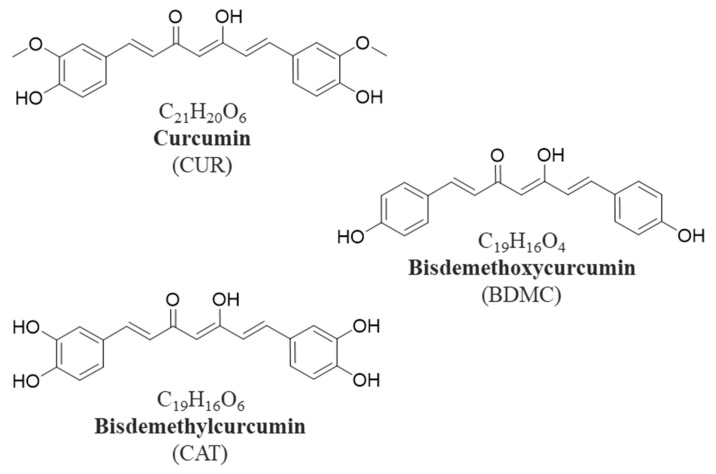
Chemical structures of curcuminoids.

Since ancient times, turmeric has been considered highly relevant, being a very versatile plant not only used for medicinal and food purposes but also in religious ceremonies, textiles and the cosmetic industry. Turmeric (*Curcuma longa*) is a herbaceous plant native to Southwest Asia and is highly valued in both Chinese and Ayurvedic medicine [1]. Among the many components that the rhizomes of the plant contain, there are proteins, fiber, minerals, vitamins, monoterpenes and sesquiterpenes, but the most important and most studied are curcuminoids, polyphenolic compounds that are responsible for the yellow-orange color of turmeric. Curcumin (CUR) (Figure 1), the main curcuminoid in turmeric, was isolated for the first time in 1815 and makes up about 75–85% of curcuminoids, followed by bisdemethoxycurcumin (BDMC) (Figure 1), demethoxycurcumin, and in minor proportion, cyclocurcumin and dihydrocurcumin [2]. Additionally, CUR is the curcuminoid that has been most widely used to evaluate its biological activity. It shows an important therapeutic potential because of its pleiotropic behavior, being able to target a wide range of molecules in different pathologies. For example, it shows antioxidant activity since it enhances the endogenous antioxidant system (superoxide dismutase, glutathione peroxidase and catalase), decreases malondialdehyde and also reduces oxidative biomarkers and lipid peroxidation [3,4].

Curcumin shows anti-inflammatory activity by binding directly to inflammatory molecules, such as tumour necrosis factor (TNF-α), cyclooxygenases COX-1 and COX-2, 5-lipoxygenase, 12-lipoxygenase, α1-human acid glycoprotein (α1-GA) and inducible nitric oxide synthase (iNOS) [5,6,7]. It has also been widely studied for the treatment of neurodegenerative diseases, such as Alzheimer’s and Parkinson’s, due to its antioxidant and anti-inflammatory properties that are capable of reducing oxidative stress (scavenging free radicals as it can donate electrons and hydrogen atoms) as well as its immunomodulatory activity. In Alzheimer’s disease, curcumin can directly bind to amyloid-β in the central nervous system and prevent its assembly into neurotoxic species. In Parkinson’s disease, it inhibits monoaminoxidase, preventing mitochondrial dysfunction, suppressing alpha-synuclein aggregation, preventing dopamine deficiency and blocking neuroinflammation [8,9,10]. Curcumin has also been used in the treatment of acquired immunodeficiency syndrome (AIDS) in conjunction with other drugs, since it acts directly on the human immunodeficiency virus HIV-1 IN and HIV-1 PR proteins, which are present in the human immunodeficiency virus (VIH), binding to them and causing their inhibition [11,12]. Many studies have shown the potent antitumor activity of curcuminoids by inhibiting the proliferation and invasion of tumors by suppressing a variety of cellular signaling pathways, inducing autophagy, apoptosis, metastasis and cell cycle arrest. This has been observed in patients with lung, colorectal, breast, brain, liver and prostate cancer [13,14,15,16,17]. It has also shown antifungal, antibacterial, hepatoprotective, immunomodulatory, hypolipidemic, hypocholesterolemic, cardioprotective, wound healing, antiplatelet and hypoglycemic activities [18,19,20,21].

The yellowish color of curcuminoids is a result of the conjugated unsaturated system, which provides a strong absorption at wavelengths around 425 nm. The unsaturated system also confers the ability to absorb light in the ultraviolet (UV) range (≈265 nm); nonetheless, other unsaturated compounds in living organisms also absorb light at this wavelength. For this reason, most methods for analyzing curcumin, especially in biological systems, use the visible range [22,23,24,25]. However, on the contrary, using the UV range may be advantageous in ex vivo permeation studies to monitor whether the skin releases any product of its structure during permeation tests. Moreover, very few works have shown the simultaneous analysis of CUR with other curcuminoids. For instance, Jadhav and co-workers, Wichitnithad and co-workers and Burapan and co-workers validated a high-pressure liquid chromatography (HPLC) method for the simultaneous analysis of CUR, BDMC and desmethoxycurcumin [23,24,26]. Nonetheless, bisdemethylcurcumin (CAT), a curcuminoid of synthetic origin, has shown better anti-inflammatory and antiproliferative properties than CUR, including the reduction in the expression of cytokine IL-6 [27], but no analytical method can be found for its analysis simultaneously with CUR and BDMC.

For these reasons, we aim at evaluating the capacity of curcumin and its derivatives (bisdemethoxycurcumin and bisdemethylcurcumin) in preventing the irritant effects of topically applied xylol; the intrinsic capacity of the curcuminoids in permeating human skin is assessed by ex vivo permeation tests. To this end, a simple, fast, selective and sensitive analytical method using HPLC in the UV range is developed and validated for the separation and quantification of CUR, BDMC and CAT in the samples from the ex vivo permeation test. This work supports future studies on the development and biopharmaceutical evaluation of dermal formulations containing these curcuminoids to determine if they exert a synergistic action.

## 2. Results and Discussion

### 2.1. Analytical Method Validation

#### 2.1.1. Linearity and Range

The linearity of an analytical procedure is its ability, within a definite range, to obtain results directly proportional to the concentration (amount) of the analyte in the sample. The linearity of the HPLC method was determined by preparing six calibration curves for the concentration range of 0.195–3.125 μg/mL for each curcuminoid.

**Figure 2:**
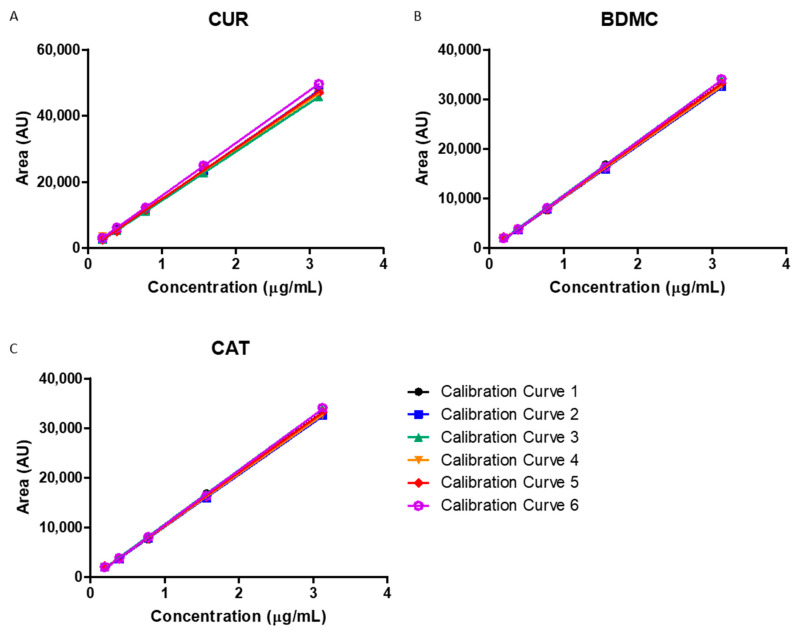
Linearity of the analytical method for curcuminoids assessed by the linear regression of the curves of (**A**) CUR; (**B**) BDMC; and (**C**) CAT (*n* = 6).

In Figure 2, the regression lines of the three curcuminoids can be observed, where their respective equations and determination coefficients (y = 15,267x − 371.2, r^2^ = 0.9999 for CUR; y = 10,720x − 357.13, r^2^ = 0.9998 for BDMC; and y = 13,313x − 428.19, r^2^ = 0.9999 for CAT) were obtained, with which it can be confirmed that the method is linear in the concentration range evaluated since coefficients of determination (r^2^) ≥ 0.99 were obtained. No statistical differences were observed after the analysis of variance (ANOVA) test in the curcuminoid calibration curves with *p*-values of 0.075, 0.523 and 0.415 for CUR, BDMC and CAT, respectively.

#### 2.1.2. Specificity

**Figure 3:**
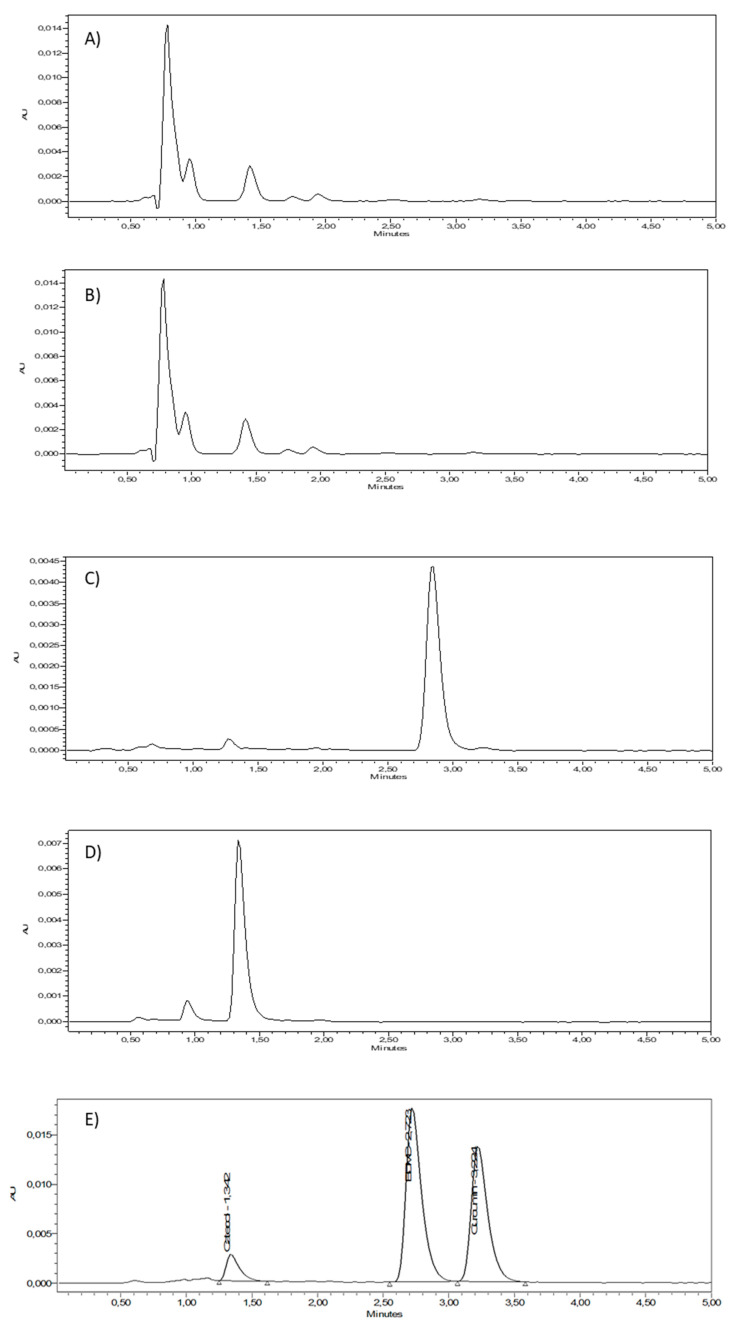

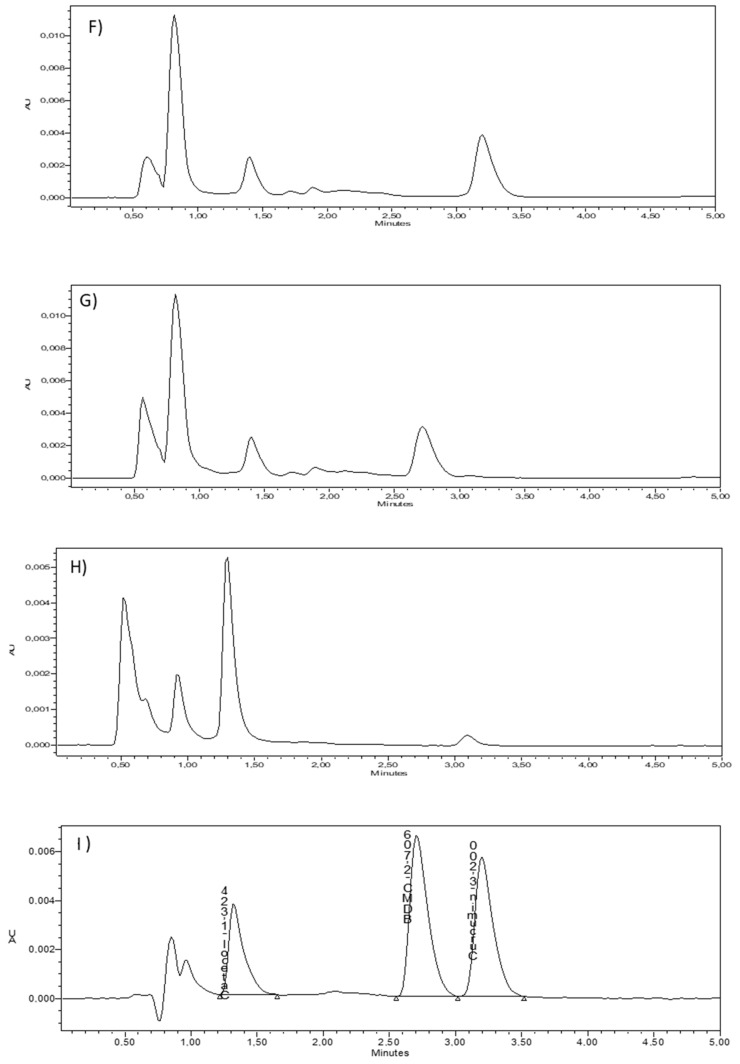
HPLC chromatograms. (**A**) blank sample (DMSO); (**B**) CUR in solution; (**C**) CUR retained in the skin; (**D**) BDMC in solution; (**E**) BDMC retained in the skin; (**F**) CAT in solution; (**G**) CAT retained in the skin; (**H**) HPLC chromatogram of the mixture of the three curcuminoids, CUR, BDMC and CAT; and (**I**) chromatogram of the three curcuminoids extracted from the skin.

Due to the multiple biological activities of curcuminoids and their low bioavailability, there is an interest in developing different formulations for their administration, especially for dermal applications. The selectivity of an analytical method is particularly relevant in the case of biological matrices, such as the skin, since it is essential to determine the analytes without any interference. Figure 3A,B shows the chromatograms of the mobile phase and dimethyl sulfoxide (DMSO), respectively, showing that at this wavelength, no relevant absorption of the mobile phase, and a little absorption of DMSO at short retention times, are observed. The chromatograms of CUR (Figure 3C), BDMC (Figure 3E) and CAT (Figure 3G) in a solution in the mobile phase, at a concentration of 3.125 µg/mL, show retention times of approximately 3.40 min for CUR, 2.85 min for BDMC and 1.35 min for CAT. The retention times observed can be thus related to the structural differences of the curcuminoids. For instance, the presence of methoxy groups in position 3 of phenyl rings in CUR is in accordance with the lower polarity and thus higher retention time, whereas the absence of a methoxy group in BDMC results in an increase in polarity and a lower retention time, and the presence of a hydroxyl group in CAT further increases the polarity and leads to the shortest retention time. Additionally, by using a short-length column (75 mm), it was possible to shorten the retention times of the compounds, which in other studies are longer.

To prove the selective detection of the curcuminoids, even in presence of other components from biological samples, the curcuminoids in solution were placed in contact with human skin for 24 h at 32 °C to promote their penetration and were then extracted again by immersing the tissue in an appropriate solvent and under sonication. DMSO was used for extracting CUR and BDMC, while the mobile phase was used for extracting CAT because DMSO shows an absorption signal close to the retention time of this compound. The chromatograms showing the individual curcuminoids CUR, BDMC and CAT extracted from the tissue can be observed in Figure 3D,F and H, respectively, showing no signals in the retention times of curcuminoids that could interfere with their quantification. Furthermore, a mixture of the three compounds was placed in contact with the skin and then extracted again to determine the specificity of the method. Once extracted from the skin, the three compounds can be separated in the column and can be observed without overlapping or additional interference. Figure 3I shows a chromatogram of the solution mixture of the three curcuminoids and Figure 3H depicts a chromatogram of the three curcuminoids extracted from the skin after being applied as a mixture to the skin.

To determine quantitatively how well the elution peaks can be differentiated in chromatographic separation, the resolution of elution was calculated. Table 1 shows the resolution for CAT–BDMC and BDMC–CUR. In both cases, the resolution was greater than 1.5, indicating that the peaks can be differentiated successfully [28].

#### 2.1.3. Sensitivity

Table 2 shows the values of the limit of detection (LOD) (confidence level of 3.3) and limit of quantification (LOQ) (confidence level of 10) according to the standard deviation of the response and the slope of the calibration curve, in which the highest sensitivity was achieved for CAT, followed by CUR and BDMC, in all cases being able to detect concentrations much lower than 1 μg/mL.

#### 2.1.4. Accuracy and Precision

The results in Table 3 show the accuracy and precision values; it can be observed that, for the three curcuminoids, the analytical method meets the acceptance criteria, since both for intra-day and inter-day repeatability the relative error (RE) and relative standard deviation (RSD) values are below the 15% allowed.

The instrumental repeatability was assessed by injecting 3.125 μg/mL of the curcuminoid solution seven consecutive times. The RSD% was 1.29%, 0.80% and 0.71% for CUR, BDMC and CAT, respectively.

#### 2.1.5. Stability

Table 4, Table 5 and Table 6 report the relative difference between the peak responses of the curcuminoid solutions at the evaluated times and the freshly prepared solutions.

The results show that the three curcuminoids are stable at room temperature and 5 °C for at least 7 days stored in amber vials. The curcuminoid solutions were also stable in the autosampler for at least 24 h. These results are in agreement with the results of Jadhav and co-workers [23], who found the curcuminoid solutions were stable for 8 weeks at room temperature and at −20 °C stored protected from light.

### 2.2. Evaluation of the Intrinsic Permeation of Curcuminoids in Ex Vivo Human Skin

**Figure 4:**
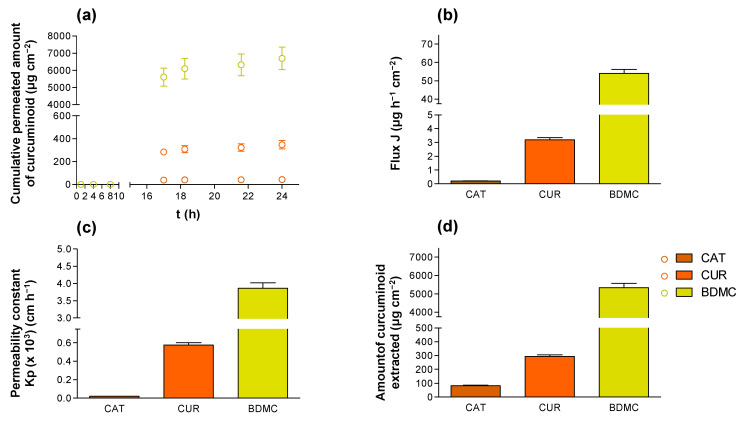
Ex vivo permeation of the curcuminoids through human skin: (**a**) permeation profile of the curcuminoids, (**b**) flux at the steady state (J), (**c**) permeability constant (Kp), (**d**) amount of curcuminoids extracted from the skin after 24-h permeation. Results represent the mean ± SD (*n* = 5).

The permeation of curcuminoids through ex vivo human skin was evaluated by means of Franz cells over 24 h. Figure 4a shows the permeation profile of the three curcuminoids, where BMDC was the curcuminoid that exhibited the greatest permeation, more than 100-fold higher than CAT and nearly 20-fold higher than CUR. BMDC had a permeation cumulative amount of 6702.3 ± 659.9 μg, followed by CUR (348.0 ± 6.0 μg) and CAT (42.7 ± 4.3 μg).

Consequently, the permeation fluxes (J) obtained for BDMC, CUR and CAT during the steady state were 54.0 ± 5.0 μg h^−1^, 3.2 ± 0.3 μg h^−1^ and 0.2 ± 0.0 μg h^−1^, respectively (Figure 4b).

As the permeation flux is directly influenced by the concentration of curcuminoid in the donor phase, to take this into consideration the permeability constant for each curcuminoid was obtained (Figure 4c), observing the same tendency.

Finally, after the 24-h permeation study, the curcuminoids were extracted from the tissue (where the possible anti-inflammatory therapeutic target can be located) using ethanol and were quantified using the validated HPLC method. A higher amount retained was observed in the case of BDMC (5344.1 ± 524.7 μg/cm^2^), followed by CUR (294.6 ± 25.7 μg/cm^2^) and CAT (7.6 ± 0.9 μg/cm^2^).

These results show that the substitution of methoxy groups of CUR in the benzene rings for hydrogen groups, leading to compound BDMC, increases its ability to penetrate into the tissue. Some proportion of the compound is then retained within the skin tissue, while another proportion can completely permeate through the tissue. The lowest permeation and retention is observed for CAT, which suggests that its higher hydrophilic behavior delays its penetration and retention through the skin, mainly of hydrophobic character as it contains hydroxyl groups instead of methoxy groups as compared to CUR.

In previous experiments, the incorporation of these curcuminoids in cationic supramolecular hydrogels leads to an electrostatic interaction between the negative charges of the beta diketone moiety of the curcuminoids and the cationic gelator, thus changing the ability of the curcuminoids to permeate through the skin tissue [29]. Specifically, their incorporation into these supramolecular gels permits penetration and retention within the tissue but prevents their complete permeation across the skin. In contrast, in this work, the curcuminoids were applied only in an ethanolic solution, which does not lead to any electrostatic interaction between the solvent and the curcuminoids. Moreover, ethanol is well-known to be a permeation enhancer. As a result, the complete permeation of the curcuminoids across the skin can be observed, as well as a greater amount retained within the tissue.

Thus, the excipients of new formulations for topical application containing curcuminoids should be selected depending on the foreseen purpose, either for achieving only local anti-inflammatory activity, where the curcuminoids should ideally only be retained within the tissue, or for achieving anti-inflammatory activity systemically, where complete permeation across the tissue is needed.

### 2.3. In Vivo Experiments

The anti-inflammatory activity of the curcuminoids in solution was assessed in rats using a xylol-induced inflammation model.

**Figure 5:**
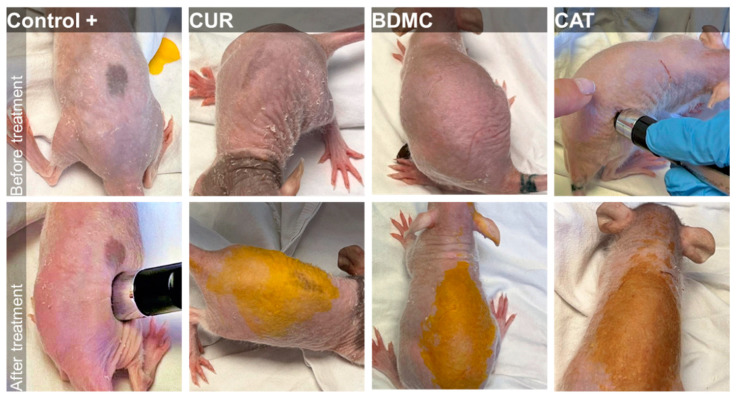
Anti-inflammatory efficacy study. Back of the rats (*n* = 6) before (upper panels) and after (lower panels) treatment with xylol and curcuminoids (CAT, CUR or BDMC). Rats treated with xylol but no curcuminoid were taken as the positive control, whereas the ventral skin of the rats was treated only with ethanol and was taken as the negative Control−.

Three groups of rats with *n* = 6 were treated as follows: Xylol was applied on the skin over the back of hairless rats together with a saturated ethanolic solution of the curcuminoids (CAT, CUR or BDMC). A fourth group of rats, treated with xylol but with no curcuminoid, was evaluated as the positive control (Control+), whereas the skin of the ventral side of the rats was treated only with ethanol and was taken as the negative control (Control−) (see Figure 5).

To the naked eye, the application of the curcuminoids prevented the irritation caused by xylol, although the strong colour of the three curcuminoids under study prevents a correct evaluation of the erythema.

**Figure 6:**
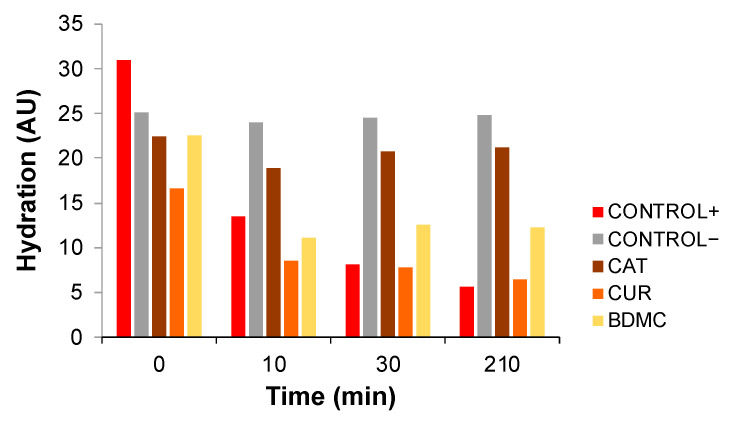
Median values of hydration (arbitrary units, AU) of the rats’ skin (*n* = 6) after treatment with xylol and curcuminoids (CAT, CUR or BDMC). Rats treated with xylol but without curcuminoid were taken as Control+, whereas the ventral skin of rats treated only with ethanol was taken as Control−.

For this reason, the hydration of the stratum corneum was assessed by a corneometer, which measures the conductivity of the skin, and therefore, reflects its hydration. Therefore, alterations in the skin barrier will result in a decrease in the hydration of the stratum corneum over time. Figure 6 shows the evolution of the stratum corneum hydration after the application of xylol as an irritant agent, followed by the application of a curcuminoid in ethanolic solution, which was compared to the application of xylol (without curcuminoid) (Control+), and to the application of only ethanol (without curcuminoid or xylol) (Control−).

The skin of rats treated with xylol (Control+) showed a slight increase in hydration immediately after application, but showed a decrease in the stratum corneum hydration up to 78% after 210 min with respect to the negative control, indicating damage to the skin barrier.

In a similar way, the treatment with CUR showed no clear prevention of this damage, resulting in a decrease in hydration equivalent to the positive control.

In contrast, the treatment with BDMC partially prevented this damage to the skin barrier, and so the hydration values only decreased partially (up to 50% after 210 min).

However, importantly, the treatment with CAT almost completely maintained the hydration of the skin (only a 16% decrease after 210 min was observed), indicating a protective role over the skin barrier from the beginning of the experiment.

### 2.4. Histology Studies

The histological study was performed at two levels: the human skin samples after ex vivo permeation experiments, and rat skin samples after in vivo experiments were observed for morphological changes at the microscopic level, for which histology experiments in both types of tissue were performed using haematoxylin and eosin staining.

**Figure 7:**
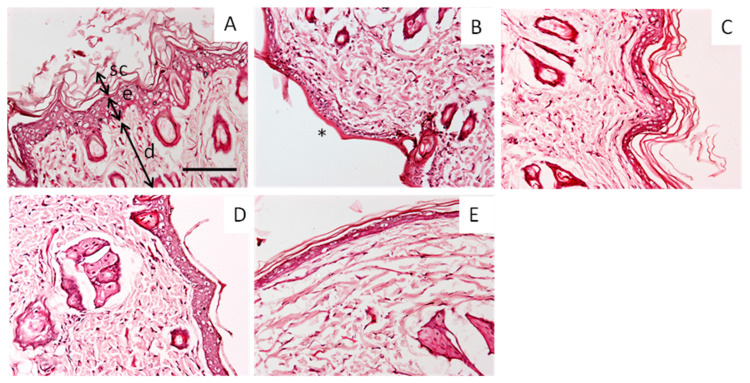
Representative histological sections (magnification 200x) of rat skin stained with haematoxylin and eosin in (**A**) control conditions (ethanol) and (**B**) upon treatment with xylol (Control+), or treated with xylol and ethanolic solution of (**C**) CAT, (**D**) CUR or (**E**) BDMC. Skin structures: sc, stratum corneum; e, epidermis; d, dermis; * indicates the loss of stratum corneum. Scale bar = 100 µm.

Figure 7 shows representative sections of rat skin stained with haematoxylin and eosin in treated with (A) ethanol (Control−) and (B) upon treatment with xylol (Control+), or treated with xylol and ethanolic solution of (C) CAT, (D) CUR or (E) BDMC.

As observed in Figure 7A, the untreated skin samples showed normal structures and appendages, including the uppermost layer and the stratum corneum, followed by the epidermis with characteristic dermal papillae, i.e., finger-like projections. A treatment with xylol was used to induce a mild irritation as a control and can be observed in Figure 7B. The xylol induced the loss of stratum corneum and reduced the presence of dermal papillae. In contrast, all three treatments decreased the alteration of the skin structures, of which CAT, shown in Figure 7C, was the most effective, preventing the effects of xylol. Its effectiveness was followed by CUR, in Figure 7D, and BDMC, in Figure 7E.

**Figure 8:**
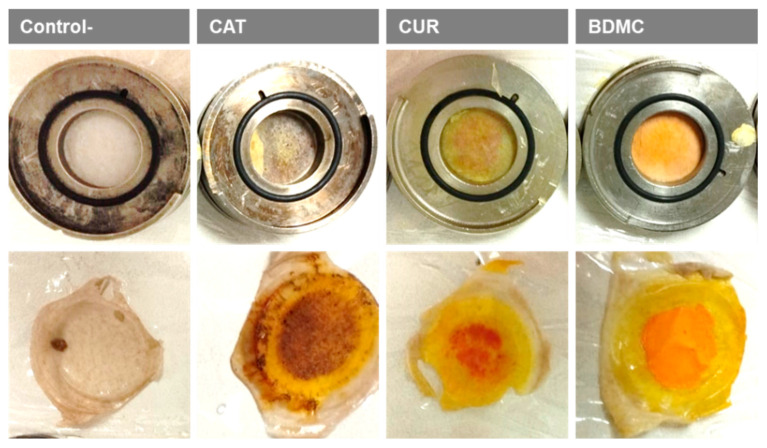
Ex vivo human skin pieces after permeation experiments with curcuminoids applied for 24 h on the skin; upper panels: mounted in the joint. The effective diffusional area was 2.54 cm^2^, lower panels: taken out of the joint. Ethanol without curcuminoid was applied as the negative control (Control−).

Additionally, the ex vivo human skin used in the permeation experiments (Section 2.2) was studied to evaluate any possible morphological changes after being in contact with the curcuminoids (Figure 8).

**Figure 9:**
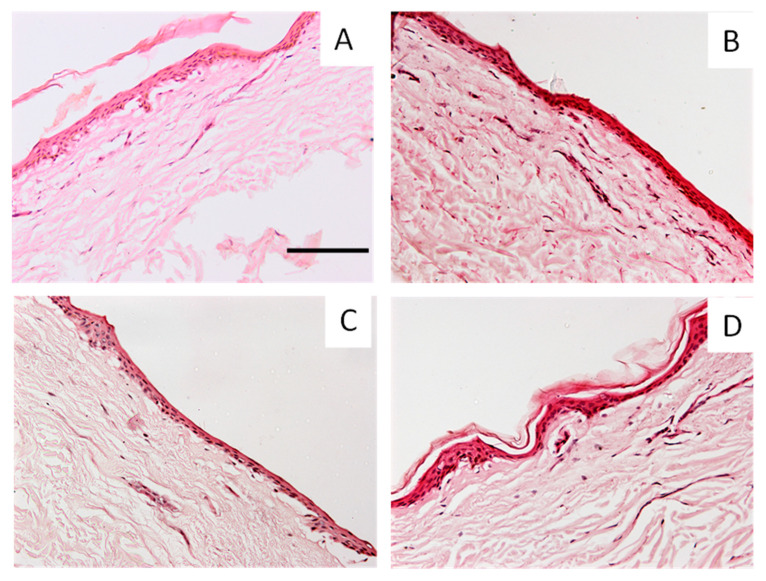
Representative histological sections of human skin stained with haematoxylin and eosin in (**A**) blank conditions (ethanol) and after being in contact with a saturated ethanolic solution of (**B**) CAT, (**C**) CUR or (**D**) BDMC. Scale bar = 100 µm.

Figure 9 shows representative sections of human skin stained with haematoxylin and eosin after being in contact with (A) ethanol (Control−) or an ethanolic solution of (B) CAT, (C) CUR or (D) BDMC.

As observed in Figure 8, the application of the formulations to the human skin did not alter its structure. The blank condition (Figure 9A) was used to see the effects of the formulations without the active ingredient. From the different curcuminoids, BDMC (Figure 9D) seems the most respectful formula to the ex vivo human skin structure, followed by CAT (Figure 9B) and CUR (Figure 9C).

## 3. Materials and Methods

### 3.1. Chemicals and Reagents

Curcumin (CUR) and bisdemethoxycurcumin (BDMC) were synthesized according to previously reported protocols [30]. Bisdemethylcurcumin (CAT) was synthesized according to a previously reported protocol [31]. The purified water used in all experiments was obtained from MilliQ^®^ Plus System (Merck, Spain), lab-supplied. All other chemicals and reagents used in the study were of analytical or HPLC grade (Fisher Scientific, Leicestershire, UK).

### 3.2. Standard Solutions for Calibration Curves

Standard stock solutions of CUR, BDMC and CAT were prepared daily by dissolving the appropriate amount of each in acetonitrile–water acidified with 2% glacial acetic acid (50:50 *v*/*v*) to obtain a final concentration of 100 µg/mL. From these solutions, dilutions were made for the calibration curves using the same solvent in the concentration range of 0.195 to 3.625 µg/mL.

### 3.3. Instrumentation and Chromatographic Conditions

To validate the methodology, High-Performance Liquid Chromatography (HPLC) from Waters was used, consisting of a Waters 515 HPLC pump, a 717 Plus autosampler and a dual λ absorbance UV-vis 2487 detector (Waters, Milford, MA, USA). The analytical chromatographic column used was a Symmetry^®^ C18, (75 × 4.6 mm and 5 µm in particle size, Waters Corporation, Spain), working at room temperature, using acetonitrile–water acidified with 2% glacial acetic acid (50:50 *v*/*v*) as the mobile phase, at isocratic conditions of elution with a flow rate of 1.2 mL/min, injection volume 10 µL and a detection wavelength of 265 nm.

### 3.4. Analytical Method Validation

The validation of the method was carried out taking into account the guidelines established by the International Conference on Harmonization (ICH) [32], and the following performance characteristics were analysed: linearity, specificity, sensitivity, accuracy and precision.

#### 3.4.1. Linearity and Range

To evaluate the linearity of the method, five concentration levels were established: 0.195, 0.391, 0.781, 1.563 and 3.125 µg/mL. The calibration curves were validated inter-day (*n* = 6, for each curcuminoid), developed by plotting the area under the curve versus the corresponding curcuminoid concentrations in a straight-line least-squares regression of the following equation: y = bx + a, where x is the concentration of the curcuminoid (µg/mL), y is the area under the curve, b is the value of the slope and a is the independent term. The least-squares fit method was employed to statistically evaluate the results for linearity by a regression line and the corresponding slope, y-intercept and coefficient of linear correlation (R^2^). Furthermore, linearity was determined by a one-way analysis of variance (ANOVA) test to compare peak areas versus nominal concentrations of each standard, and differences were considered statistically significant when *p* < 0.05. The least-square linear regression analysis and mathematical determinations were performed by the Prism^®^, V. 5.01 software (GraphPad Software Inc., San Diego, CA, USA).

#### 3.4.2. Specificity

Specificity is the ability of an analytical method to assess unequivocally the analyte in the presence of components that may be expected to be present. In this validation, specificity was assessed by determining: (a) the individual curcuminoid standard solution, (b) a mixture of the three curcuminoids in the dissolution medium (mobile phase), (c) the individual samples from drug extraction from the skin, and finally (d) a mixture of the three curcuminoids from drug extraction from the skin. The standard solutions of the curcuminoids were prepared at a concentration of 3.125 µg/mL. The curcuminoid-extracted samples were obtained by immersing a skin disc in the corresponding curcuminoid or mixture of curcuminoids for 24 h, following a drug extraction procedure (see Section 3.5).

To verify that the method would not have interferences when analysing the analytes of interest in the biological samples, the chromatograms were reviewed to evaluate whether the peaks of the different curcuminoids or any component from the skin eluted at different retention times. Additionally, the goodness of the specificity was also evaluated by calculating the peak resolution according to the following equation:

(1) $$R_{s}=\frac{2\cdot \left[{tr}_{B}-{tr}_{A}\right]}{W_{B}+W_{A}}$$

where *R_s_* is the resolution of the elution between two consecutive peaks, and *tr_A_* and *tr_B_* are the retention times of the first and second component’s peak, respectively. *W_A_* and *W_B_* are the widths of the respective components’ peak [28].

#### 3.4.3. Sensitivity

The sensitivity of the method was determined concerning the limit of detection (LOD) and limit of quantification (LOQ), being the lowest concentration of curcuminoids in a sample that can be detected or quantified, respectively, with the criteria of accuracy and precision. Statistically, the two parameters are characterized by being theoretically calculated taking into account the slope, the intercept and the standard deviation of the residuals obtained in the first-order linear regression of the linearity of the system with a confidence of 95% using the following equation:

(2) $$\mathrm{LOD}\;\mathrm{or}\;\mathrm{LOQ}\;=k\times \frac{{SD}_{Sa}}{Sb}$$

where *k* is the factor related to the level of confidence. Its value is 3.3 for LOD and 10 for LOQ, and *SD_Sa_* is the standard deviation of the intercept and *S_b_* is the slope.

#### 3.4.4. Accuracy and Precision

Accuracy and precision were calculated by analysing samples at three concentration levels: low, medium and high for each curcuminoid (0.195, 0.781 and 3.125 μg/mL, respectively). The accuracy of the method was evaluated according to the relative error (*RE*, %) (Equation (3)), the ratio of measured concentrations with respect to the nominal concentrations. The precision was determined by the intra-day and inter-day repeatability; different samples of the three curcuminoids were analysed at the three concentration levels on the same day (intra-day) and during different days (inter-day) in three replicates each. Finally, the instrumental repeatability was evaluated by analysing the same sample (3.125 μg/mL), repeatedly, 7 consecutive times for each curcuminoid. The precision data were expressed as the relative standard deviation (*RSD*, %) (Equation (4)). The method is considered accurate and precise if the RE and RSD values are within ±15% [32].

(3) $$RE(\%)=\frac{V_{a}-V_{e}}{V_{e}}\times 100$$

(4) $$RSD\left(\%\right)=\frac{s}{\overset{-}{x}}\times 100$$

where RE is the relative error, $V_{a}$ is the approximate o measured value, $V_{e}$ is the exact or nominal value, RSD is the relative standard deviation, s is the standard deviation and $\overset{-}{x}$ is the sample mean.

#### 3.4.5. Stability

In this validation, the stability of the curcuminoids in the standard solutions was assessed at room temperature and at 5 °C for 24 h, 48 h, 5 days and 7 days, and in the autosampler for 24 h. The standard solutions were prepared at the concentration of 3.125 µg/mL and stored in amber vials. The relative difference (Equation (5)) between the area response in the freshly prepared standard solution and the standard solution stored at room temperature, fridge or in the autosampler was calculated for each stability time point. The solutions were considered stable if the relative difference between the curcuminoid responses was ≤3.0%.

(5) $$Rel.Diff.(\%)=\frac{T_{x}-T_{0}}{T_{0}}\times 100$$

where *T_x_* is the area response of the curcuminoid peak at a particular time point and *T*_0_ is the area response of the curcuminoid peak at time 0, which was freshly prepared.

### 3.5. Evaluation of the Intrinsic Permeation of Curcuminoids in Ex Vivo Human Skin

To test the amount of curcuminoid that can permeate the human skin and be retained within the tissue, permeation experiments were performed using Franz diffusion cells. Human skin with a thickness of 0.4 mm was used, obtained from the abdominal region during plastic surgery of a healthy 40-year-old woman who provided written informed consent to the use of this material for research purposes with the approval of the Ethics Committee. The skin was placed between the receptor and the donor compartments with the epidermal side of the skin facing the donor compartment. The system was fixed using Parafilm^®^ (Sigma-Aldrich, Madrid, Spain) and clamps to prevent any leakage or solvent evaporation. The receptor compartment was filled with ethanol, complying sink conditions. The Franz cells were placed under a circulating water bath at 32 °C. A total of 1 mL of an ethanolic saturated solution of curcuminoid (BDMC, CUR or CAT) was seeded over the epidermal side of the skin in the donor compartment, and samples were taken over 24 h, which were kept at −20 °C until analysis using the validated HPLC method.

After the experiment, the skin was washed with a 0.05% sodium lauryl sulphate solution and rinsed thoroughly with deionized H_2_O. To extract the curcuminoids from the skin, the effective diffusion area of the skin was cut, weighed and punctured with a needle to increase the surface area. Afterward, the skin was immersed in 1 mL ethanol and samples were immersed in a sonication bath for 20 min. The skin was removed, and the solutions with the curcuminoid extracted were kept at −20 °C until analysis using the validated HPLC method.

### 3.6. In Vivo Experiments

The anti-inflammatory activity of the curcuminoid solutions was assessed in rats using a xylol-induced inflammation model. Four groups of rats (*n* = 6) were treated as follows: Group 1 (positive control): an ethanolic solution of xylol was applied on the skin over the back of hairless rats to induce inflammation; Group 2: an ethanolic solution of xylol was applied on the skin over the back of hairless rats to induce inflammation and 5 minutes later a saturated ethanolic solution of CUR; Group 3: an ethanolic solution of xylol was applied on the skin over the back of hairless rats to induce inflammation and 5 minutes later a saturated ethanolic solution of BDMC; and Group 4: an ethanolic solution of xylol was applied on the skin over the back of hairless rats to induce inflammation and 5 minutes later a saturated ethanolic solution of CAT. The ventral side of the rats was treated only with ethanol and was taken as a control.

The efficacy was assessed by means of the hydration of the stratum corneum, by measuring the conductivity of the skin with the help of a corneometer. The median hydration values (AU) of each group at certain intervals were plotted.

### 3.7. Histology Studies

Human skin samples after ex vivo permeation experiments and rat skin samples after in vivo experiments were observed for morphological changes at the microscopic level, for which histology experiments in both types of tissue were performed using hematoxylin and eosin staining.

The skin samples were fixed in 4% buffered formaldehyde at room temperature for 24 h, washed in PBS for 3 h, and then dehydrated in graded ethanol (70%, 90% and 100%), and the samples were finally embedded in paraffin. The paraffin-embedded samples were cut into 5 μm sections and stained with hematoxylin and eosin. Afterward, the samples were analyzed under a microscope Olympus BX41 and Olympus XC50 camera(Olympus, Barcelona, Spain) with 200 times magnification for the evaluation of the tissue structure.

## 4. Conclusions

An analytical method for quantifying the curcuminoids was validated within the range of 0.195–3.125 μg/mL for the three curcuminoids. The three curcuminoids were stable at room temperature and 5 °C for at least 7 days when stored in amber vials.

The intrinsic permeation of curcumin, bisdemethoxycurcumin and bisdemethylcurcumin was evaluated by ex vivo permeation tests. All three curcuminoids were able to diffuse across the skin, with outstanding permeation and retention in the skin found for bisdemethoxycurcumin (BDMC).

The application of the curcuminoids to rats prevented the degradation of the skin barrier function caused by xylol, as it was able to maintain the hydration of the skin, with special efficacy observed for CAT. Finally, the histological analysis showed that all three treatments decreased the alteration of the skin structures, with CAT being the most effective curcuminoid in preventing the morphological alterations of the skin at the microscopic level induced by xylol.

## Figures and Tables

**Table 1:** Peak resolution of two consecutive curcuminoids peaks.

	Resolution of the Elution
Rs _(CAT–BDMC)_	1.51
Rs _(BDMC–CUR)_	3.89

Rs stands for resolution.

**Table 2:** Detection and quantification limits of the analytical method for curcuminoids.

	CUR	BDMC	CAT
LOQ (μg/mL)	0.13 ± 0.09	0.17 ± 0.06	0.09 ± 0.06
LOD (μg/mL)	0.04 ± 0.03	0.06 ± 0.02	0.03 ± 0.02

Values represent means ± SD. LOQ = Limit of quantification; LOD = Limit of detection.

**Table 3:** Precision and accuracy of the HPLC method for the determination of curcuminoids.

		CUR			BDMC			CAT		
	Level (µg/mL)	Recovery (µg/mL)	RSD (%)	RE (%)	Recovery (µg/mL)	RSD (%)	RE (%)	Recovery (µg/mL)	RSD (%)	RE (%)
Intra-day (*n* = 3)	3.125	3.118 ± 0.01	0.35	0.23	3.128 ± 0.01	0.39	−0.08	3.134 ± 0.01	0.18	−0.28
	0.781	0.779 ± 0.01	0.65	0.24	0.760 ± 0.02	2.29	2.78	0.778 ± 0.01	1.91	0.41
	0.195	0.210 ± 0.02	9.83	−7.53	0.219 ± 0.00	2.02	−12.36	0.211 ± 0.01	5.24	−8.20
Inter-day (*n* = 6)	3.125	3.131 ± 0.00	0.11	−0.18	3.137 ± 0.01	0.19	−0.38	3.128 ± 0.01	0.24	−0.11
	0.781	0.775 ± 0.01	1.22	0.84	0.767 ± 0.01	1.09	1.86	0.776 ± 0.01	1.45	0.68
	0.195	0.209 ± 0.01	2.94	−6.84	0.222 ± 0.00	1.30	−13.49	0.199 ± 0.01	5.69	−1.88

Recovery = Measured concentrations. Values represent means ± SD; RSD = Relative standard deviation; RE = Relative error.

**Table 4:** Results obtained for the stability of the curcuminoid solutions stored at room temperature for 7 days. The stability was considered as the relative difference of the area response of 3.125 µg/mL of curcuminoid solution between each stability time point and the freshly prepared solutions.

	Relative Difference (%)		
Time	CUR	BDMC	CAT
Time 1 (days)	0.44	0.77	−0.45
Time 2 (days)	−0.14	0.31	−0.45
Time 5 (days)	0.30	−0.06	−0.96
Time 7 (days)	0.75	−0.57	−0.70

**Table 5:** Results obtained for the stability of the curcuminoid solutions stored at 5 °C for 7 days. The stability was considered as the relative difference of the area response of 3.125 µg/mL of curcuminoid solution between each stability time point and the freshly prepared solutions.

	Relative Difference (%)		
Time	CUR	BDMC	CAT
Time 1 (days)	0.10	0.73	−0.50
Time 2 (days)	−0.12	−0.08	−0.61
Time 5 (days)	−0.63	−0.19	0.72
Time 7 (days)	0.04	0.63	0.83

**Table 6:** Results obtained for the stability of the curcuminoid solutions stored in the autosampler (room temperature) for 24 h. The stability was considered as the relative difference of the area response of 3.125 µg/mL of curcuminoid solution between the freshly prepared solutions and 24 h after storage in the autosampler.

	Relative Difference (%)		
Time	CUR	BDMC	CAT
Time 1 (days)	−0.50	−0.94	−0.50

## Data Availability

Not applicable; the data are included within this article.
